# Microglial Activation Plays a Critical Role in LPS‐Induced Contextual Fear Memory Consolidation Impairment and Blood‐Brain Barrier Disruption

**DOI:** 10.1002/brb3.71747

**Published:** 2026-09-06

**Authors:** Jun Zheng, Linxuan Li, Xinchun Jin, Wencao Liu

**Affiliations:** ^1^ Department of Medicine Shanxi Health Vocational College Shanxi China; ^2^ Department of Histology and Embryology School of Basic Medical Sciences Advanced Innovation Center for Human Brain Protection Capital Medical University Beijing China; ^3^ Department of Emergency Shanxi Provincial People's Hospital Taiyuan China

**Keywords:** blood‐brain barrier, contextual fear memory, hippocampus, LPS, microglia

## Abstract

**Background:**

Accurately predicting environmental dangers is a fundamental ability for animal and human survival. Fear memory could be disrupted by various stimuli, including inflammation, which can be mimicked by lipopolysaccharide (LPS) injection. LPS, which can activate microglia and disrupt the blood‐brain barrier (BBB), can impair contextual fear memory consolidation when injected immediately after fear conditioning. However, the mechanism is not clear. Minocycline, a tetracycline derivative, can reduce inflammation and microglial activation. This study aims to explore the role of microglial activation in LPS‐induced contextual fear memory consolidation impairment and BBB disruption.

**Methods:**

Immediately after the fear conditioning training, 0.5 or 1 mg/kg LPS was injected i.p., and the contextual fear memory was tested 24 h later, followed by biochemical and histological test.

**Results:**

The results showed that LPS impaired contextual fear memory consolidation, which was accompanied by a significant leakage of immunoglobulin G (IgG) in the hippocampus 24 h after LPS treatment. In addition, significantly decreased levels of claudin‐5 and occludin, and significantly upregulated levels of caveolin‐1, microglial activation, and the levels of pro‐inflammatory factors interleukin‐1β (IL‐1β) and tumor necrosis factor‐α (TNF‐α) were found in the hippocampus. In contrast, minocycline, which could inhibit microglial activation, reduce LPS‐induced memory impairment and BBB damage.

**Conclusions:**

In summary, the study provides preliminary evidence that LPS can impair the consolidation of contextual fear memory by causing microglial activation, releasing pro‐inflammatory factors IL‐1β and TNF‐α, and leading to BBB disruption in the hippocampus of mice.

## Graphical abstract

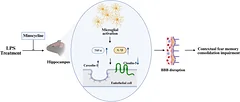

**Abbreviations**
BBB: blood‐brain barrier
CRTC1: CREB‐regulated transcription coactivator 1
CS: conditioned stimulus
IgG: immunoglobulin G
IL‐1β: nterleukine‐1β
LPS: lipopolysaccharide
TNF‐α: tumor necrosis factor‐α
US: unconditioned stimulus

## 1 Introduction

Accurately predicting environmental damages is an essential ability for animals and humans to survive, and if the memory of fear is impaired, animals and humans lose the ability to avoid danger (Vasudevan et al. 2024). Newly acquired memories undergo a series of processes at the molecular and cellular levels to become stable, a process known as memory consolidation, which typically occurs within a few hours of memory learning (Haubrich et al. 2020). In the initial stage of consolidation, the stability of newly formed memories is insufficient, and they are easily disturbed and interrupted by various external or internal factors (Haubrich et al. 2020).

Fear memory could be impaired by various stimuli, including inflammation (Davis et al. 2026), and neuroinflammation plays an important role in the pathogenesis of cognitive dysfunction (Skelly et al. 2018; Wei et al. 2015). Systemic inflammation, which releases lipopolysaccharide (LPS) into circulation (Shukla et al. 2014), can cause emotional and cognitive dysfunction through the release of a substantial quantity of inflammatory factors and the activation of immune cells (Vasconcelos et al. 2014). Immediately after fear memory training, LPS injection can impair the memory consolidation process (Haskel et al. 2026). However, the mechanism by which LPS induces fear memory deficiency is unclear, there is currently no effective strategy for inflammation‐induced fear memory deficiency.

Contextual fear memory is dependent on the hippocampus, and clinical studies have shown that disruption of the hippocampal blood‐brain barrier (BBB) is an important feature in patients with early cognitive dysfunction (Montagne et al. 2015). LPS can impair the integrity of the BBB in depressive‐like behavior (Cao et al. 2025), which is mainly composed of cerebral microvascular endothelial cells and tight junctions between the endothelial cells (Y. P. Wang, Du, Hu, et al. 2023). In addition, LPS can activate microglial cells to release pro‐inflammatory factors TNF‐α and IL‐1β (Shen et al. 2018). Microglial activation precedes BBB destruction and cell infiltration, and can lead to disruption of BBB integrity (Chen et al. 2022; Zhang et al. 2024), leading to cognitive impairment (Eyvari‐Brooshghalan et al. 2025). Caveolin‐1‐mediated BBB disruption via matrix metalloproteases 2/9 contributes to postoperative cognitive dysfunction (Li et al. 2025). The dysfunction of parvalbumin interneurons mediated by microglia contributes to cognitive impairment induced by LPS challenge (Mao et al. 2021). However, it is unclear whether microglia‐induced BBB damage is associated with the disruption of contextual fear memory consolidation.

Minocycline is a tetracycline antibiotic. It can cross the BBB and attenuate inflammation associated with microglial activation (G. Wang et al. 2019), attenuate LPS‐induced cognitive impairment in mice (Hou et al. 2016), and attenuate brain injury caused by transient focal cerebral ischemia due to degradation of the tight junction protein occludin (X. Jin et al. 2013). Therefore, in the current study, minocycline is used to check the effect of inflammation on LPS‐induced memory impairment and BBB disruption.

In animal models, fear learning is typically achieved through a Pavlovian learning behavioral paradigm between a conditioned stimulus (CS)—that is, an environment or environmental change—and an unconditioned stimulus (US)—that is, one or more foot shocks (X. C. Jin et al. 2007). The fear conditioning task is an ideal behavioral paradigm for studying fear memory.

In our current study, we will use the fear conditioning task to test our hypothesis that microglial activation plays a critical role in LPS‐induced contextual fear memory consolidation impairment and BBB disruption.

## 2 Materials and Methods

### 2.1 Animal

Male C57BL/6 mice (8–10 weeks) were obtained from Beijing Vital River Laboratory Animal Technology Co. Ltd. They were raised in a specific pathogen‐free (SPF) environment with constant temperature (23 ± 1°C) and were provided with ad libitum access to food and water. All experimental designs were approved by the Animal Care Committee of Capital Medical University and were carried out following the NIH Guide for the Care and Use of Laboratory Animals. All designs followed the principle of reducing and minimizing the number of animals and their suffering.

### 2.2 Drugs and Drug Administration

LPS (Sigma, St. Louis, MO, USA) was dissolved in sterile saline and intraperitoneally (i.p.) injected immediately after training at a dose of 0.5 or 1 mg/kg.

Minocycline was dissolved in saline. Pretreatment with minocycline at a dose of 50 mg/kg for three consecutive days. Two hours after injection, mice received training, and immediately after training, LPS was administered at a dose of 0.5 mg/kg.

### 2.3 Behavior Test: Fear Conditioning

#### 2.3.1 Behavioral Procedures

For fear conditioning, mice were placed in a chamber and allowed to explore it for 2 min. Then, a tone (30 s, 2000 Hz, and 80 dB) was given (CS). The last 1 s of the CS was paired with a foot shock (0.5 mA; US). Mice received three CS–US pairs with an inter‐CS interval of 30 s. Mice were allowed to stay in the chamber for an additional 1 min. Then, they were returned to their home cages. For testing, mice were placed in the chamber for 3 min without tone. Freezing score was used as a behavioral measure of fear memory.

##### 2.3.1.1 Measurement of Locomotor Activity

The behavioral response of mice in the open field box test was recorded by a camera and a computer connected to it with Any‐maze software (Shanghai Xinruan Information Technology Co. Ltd). Based on the time taken by the mice and the distance traveled, speed was calculated.

##### 2.3.1.2 Experimental Procedures

###### 2.3.1.2.1 Experimental 1

**FIGURE 1:**
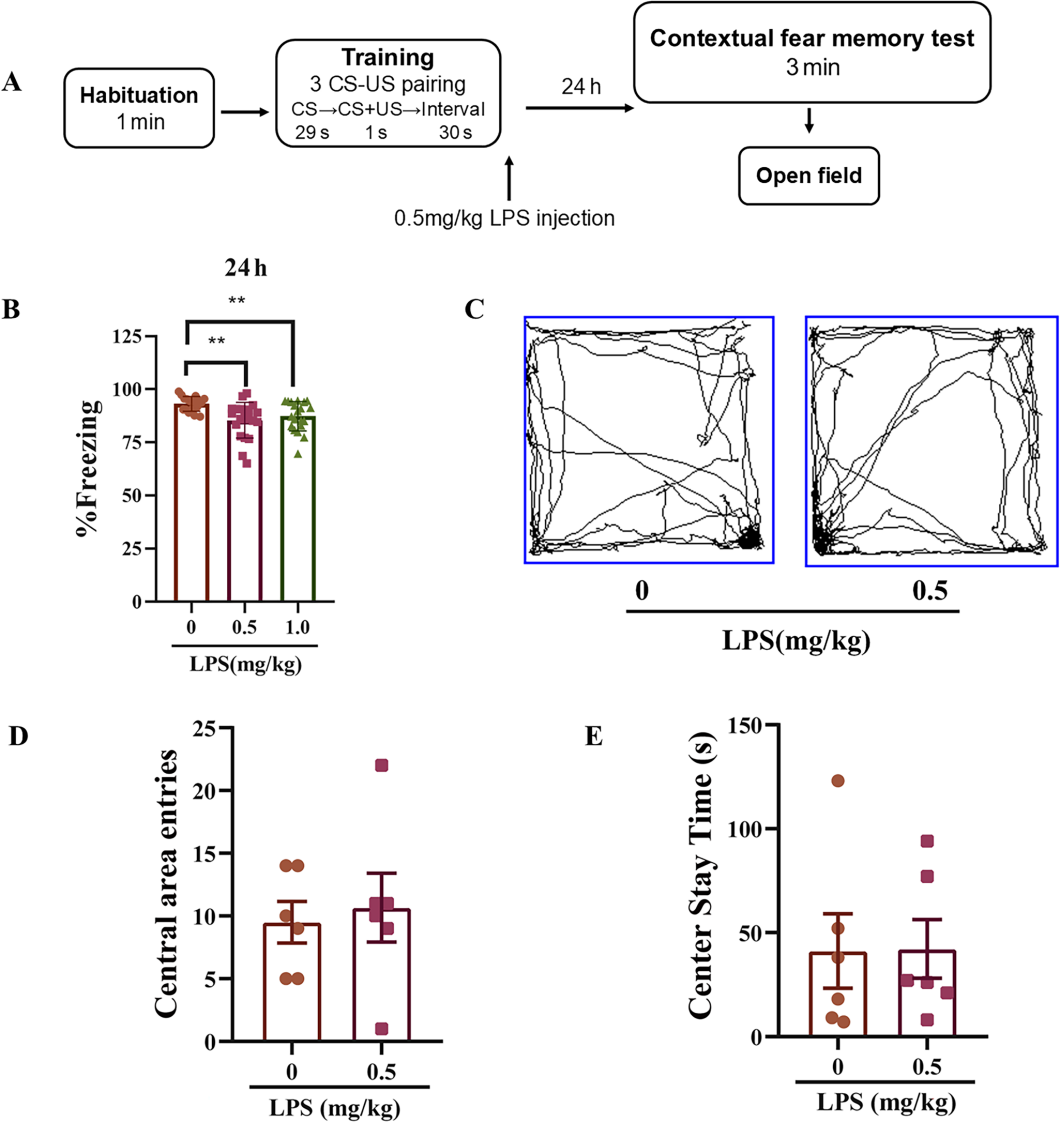
LPS‐induced impairment of scene fear memory consolidation in mice. (A) The diagram illustrates the procedure in the experiment. (B) Contextual fear memory levels were detected 24 h after intraperitoneal injection of saline (*n* = 17), 0.5 mg/kg (*n* = 19), and 1.0 mg/kg (*n* = 20) LPS. *p < 0.05, **p < 0.01 vs. the Ctrl group. (C–E) Open field experiments were performed 24 h after LPS treatment. There was no significant difference between the groups for the total distance traveled (C), number of entries in the central region (D), or the central stay time (E). *n* = 6/group.

This test aimed to examine the impact of LPS treatment on contextual fear memory consolidation deficiency. Mice were randomly divided into three groups: Saline, LPS (0.5 mg/kg), and LPS (1 mg/kg) (see Figure 1A for detailed experimental procedures).

###### 2.3.1.2.2 Experimental 2

This test aimed to examine the impact of LPS treatment on BBB permeability and microglial activation in the hippocampus.

###### 2.3.1.2.3 Experimental 3

This test aimed to examine the effect of minocycline on LPS‐induced impairment of fear memory. Mice were randomly assigned to LPS (0.5 mg/kg) or minocycline (50 mg/kg)+LPS (0.5 mg/kg) (see Figure 3A for detailed experimental procedures).

###### 2.3.1.2.4 Experimental 4

This test aimed to examine the effect of minocycline on LPS‐induced BBB damage and microglial activation in the hippocampus. Mice were randomly assigned to LPS (0.5 mg/kg) or minocycline (50 mg/kg)+LPS (0.5 mg/kg). (see Figure 3A for detailed experimental procedures).

### 2.4 Evaluation of BBB Permeability by Detection of Immunoglobulin G Leakage

Immunoglobulin G (IgG) leakage was used to evaluate BBB permeability as we described previously (X. Wang et al. 2016). To measure the extravasation of endogenous IgG, the 20 µm thick brain sections were blocked in 5% (wt/vol) BSA (A8020, Solarbio, Beijing, China) for 2 h. Sections were then incubated with Cy‐3‐conjugated Affinity Pure Goat anti‐Mouse IgG (1:500; A11001, Invitrogen, Carlsbad, USA) at room temperature for 2 h. The slide was scanned in an LSM 700 microscope (Carl Zeiss).

### 2.5 Immunoblotting for Occludin and Caveolin‐1

Mice received an overdose of anesthesia with pentobarbital and were perfused with pre‐cooled PBS after the behavioral test (Hu et al. 2024). The expression levels of occludin and caveolin‐1 in the mouse hippocampus were analyzed. Tissues were homogenized and lysed in a lysis buffer (50 mM Tris‐HCl, 150 mM NaCl, 5 mM CaCl_2_·2H_2_O, 1% Triton X‐100, and 0.05% Brij‐35, pH 7.6). The protein concentration was measured using a BCA protein assay kit (Beyotime, Haimen, China). Equal amounts (30 µg of total protein) of each homogenate aliquot were boiled at 100°C for 5 min, and then the proteins were separated by electrophoresis in 10% or 12.5% SDS‐PAGE gels, transferred onto PVDF membranes (0.45 µm, Millipore, Billerica, USA). Membranes were subsequently blocked in TBS‐T (Tris‐buffered saline and 0.1 % Tween‐20) containing 5 % nonfat milk for 1 h and incubated with primary antibodies: occludin (1:500; Invitrogen, USA), caveolin‐1 (1:1000; CST), or GAPDH (1:5000; Boster, China) at 4°C for 12 h. After TBS‐T washing, the membranes were incubated with the corresponding secondary antibodies (Boster, China) for 2 h. Finally, the membranes were subjected to incubation with the SuperSignal West Pico HRP substrate reagents (Thermo Fisher, USA) and image capture. The protein expression levels of occludin and caveolin‐1 were quantified using ImageJ software and normalized to GAPDH as the loading control.

### 2.6 Immunofluorescence Staining for Claudin‐5, CD31, Iba‐1

Immediately after the behavioral tests, mice received an overdose of anesthesia with pentobarbital and were subsequently perfused with PBS and fixed with 4% PFA. The brain tissues were then sectioned into 20‐µm‐thick brain cryosections for the evaluation of claudin‐5, CD31, and Iba‐1, as previously described (Q. Wang, Wang, et al. 2023). Briefly, the nonspecific component was removed with a blocking buffer containing 0.3% Triton X‐100, 1% BSA, and 5% goat serum. The blocked sections were incubated first with primary antibodies against claudin‐5 (1:200, Invitrogen, USA), CD31 (1:200; R&D), or Iba‐1 (1:200, Millipore) overnight at 4°C, and then with the secondary antibody (anti‐rabbit Alexa Fluor 488‐conjugated, diluted at 1:800) for 1 h at room temperature. The staining of hippocampus sections was captured using a Zeiss LSM 700 microscope.

### 2.7 Real‐Time RT‐PCR

Total RNA was extracted from brain tissue using the TRIzol reagent (Invitrogen, USA) according to the manufacturer's instructions. cDNA synthesis was then performed with 3 µg of total RNA and random primers using TaqMan Reverse Transcription Kits (Applied Biosystems) in a 20 µL reaction volume. The reaction mixture was incubated at the appropriate temperature and time as specified in the kit protocol to complete reverse transcription. The quantification primers (synthesized by Integrated DNA Technologies) used in this study were designed according to the known mouse sequences: IL‐1β forward: 5’‐TGGAAAAGCGGTTTGTCTTC‐3’, reverse:5’‐TACCAGTTGGGGAACTCTGC; TNF‐α forward:5’‐CATCTTCTCAAAATTCGAGTGACAA‐3’, reverse:3’‐TGGGAGTAGACAAGGTACAACCC‐5’; GAPDH forward: 5’‐CAATGTGTCCGTCGTGGATCT‐3’, reverse:3’‐GTCCTCAGTGTAGCCCAAGATG‐5’. The SDS Enterprise Database software (Applied Biosystems) was employed for computing the fluorescence threshold cycle (Ct) value. Fold changes in mRNA expression were determined by ΔΔCt, as previously described. and expressed as 2‐ΔΔCt values (X. Wang et al. 2024).

### 2.8 Statistical Analysis

Formal tests for normality were used to assess data distribution using the Shapiro–Wilk test. Data are shown as the mean ± SEM. Statistical analysis was performed using the SPSS 18.0 software (SPSS, Chicago, IL, USA). One‐way ANOVA, followed by Bonferroni post hoc tests, was used for data analysis. *p* < 0.05 was considered statistically significant.

## 3 Results

### 3.1 LPS‐Induced Contextual Fear Memory Consolidation Impairment in Mice

LPS and cytokines can impair memory consolidation and reconsolidation (Haskel et al. 2026; Shu et al. 2020). Figure 1A shows the experimental procedure. As shown in Figure 1B, immediate post‐conditioning i.p. LPS treatment at doses of 0.5 and 1.0 mg/kg significantly impaired fear memory, which was detected 24 h later. A 0.5 mg/kg dose was chosen for intraperitoneal injection in future experiments. In addition, an open‐field test was performed 24 h after LPS injection, and it was found that there was no significant difference in total distance traveled, the number of times the mice crossed the central zone, and the residence time in the central area between the three groups (Figure 1C–E), suggesting that LPS injection does not affect the voluntary activities of mice.

### 3.2 LPS‐Induced BBB Hyperpermeability in the Hippocampus in Mice

**FIGURE 2:**
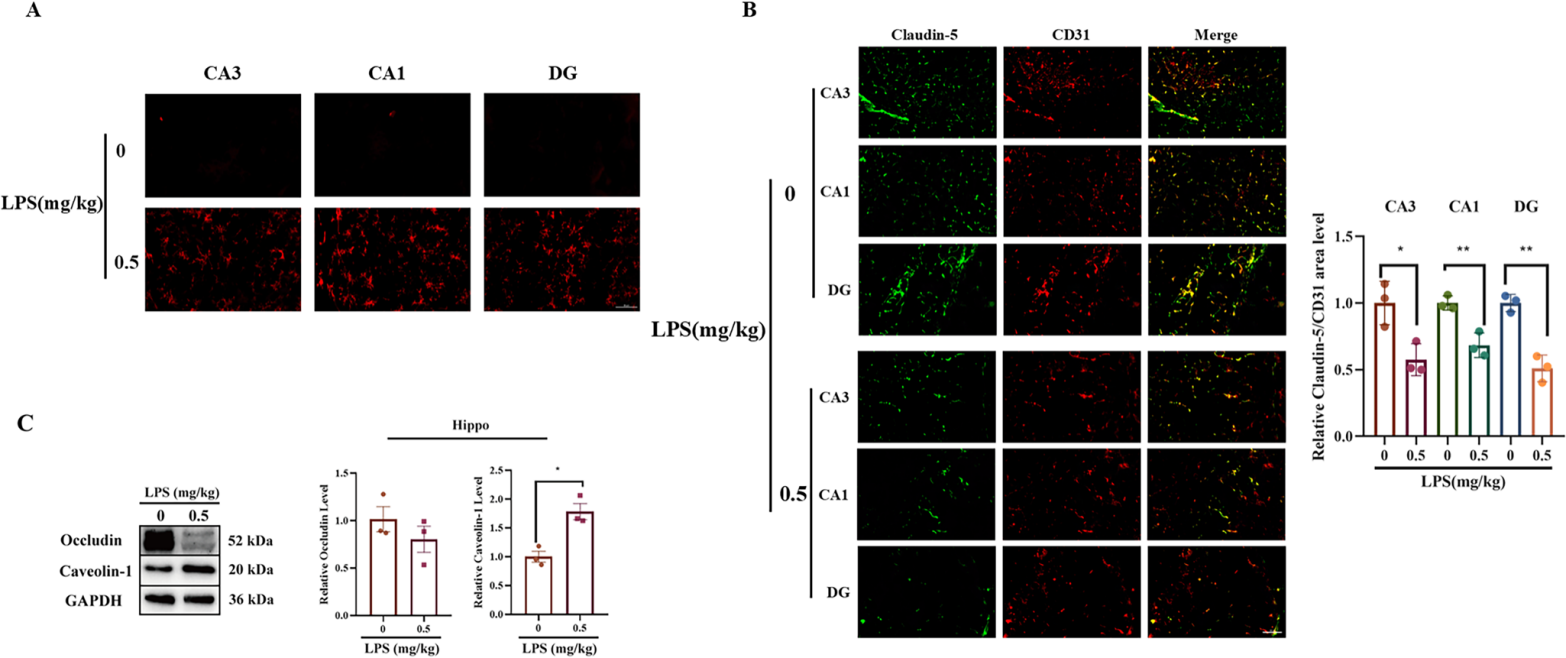
LPS induces blood‐brain barrier hyperpermeability in the hippocampus in mice. (A) Immunofluorescence showed the leakage of IgG in hippocampal CA3, CA1, and DG brain regions after LPS treatment, *n* = 3/group. Scale bar = 50 µm. (B) In the left panel, representative immunofluorescence results showed the expression of claudin‐5 and CD31 in the hippocampal CA3, CA1, and DG 24 h after LPS treatment. Scale bar = 100 µm. In the right panel, relative claudin‐5/CD31 area was quantified. (C) Representative Western blot results showed occludin and caveolin‐1 expression after LPS treatment. The relative band intensity was quantified (*p < 0.05 vs. vehicle, **p < 0.05 vs. the Ctrl group, *n* = 3/group).

Tight junction proteins occludin and claudin‐5 are the key structural components of the BBB (Y. Wang, Du, Sun, et al. 2023), and caveolin‐1 can modulate the permeability of the BBB (Andreone et al. 2017). BBB permeability was examined by detecting occludin, claudin‐5, caveolin‐1, and IgG after the behavioral test. Immunofluorescence results showed that LPS caused significant IgG leakage (Figure 2A) in CA3, CA1, and DG regions of the hippocampus. Co‐staining of claudin‐5 and CD31 was done to show the distribution of claudin‐5 in the hippocampus. As depicted in Figure 2B, claudin‐5 was found in blood vessels in the hippocampus, and LPS treatment notably downregulated the expression of claudin‐5 and CD31 in the hippocampus. The WB results showed that the level of caveolin‐1 was significantly increased (Figure 2C), and the level of occludin was decreased in the hippocampus of mice, indicating that LPS can lead to an increase in the permeability of the hippocampal BBB in mice.

### 3.3 LPS Promotes Microglial Activation and Release of Pro‐Inflammatory Factors From the Mouse Hippocampus

**FIGURE 3:**
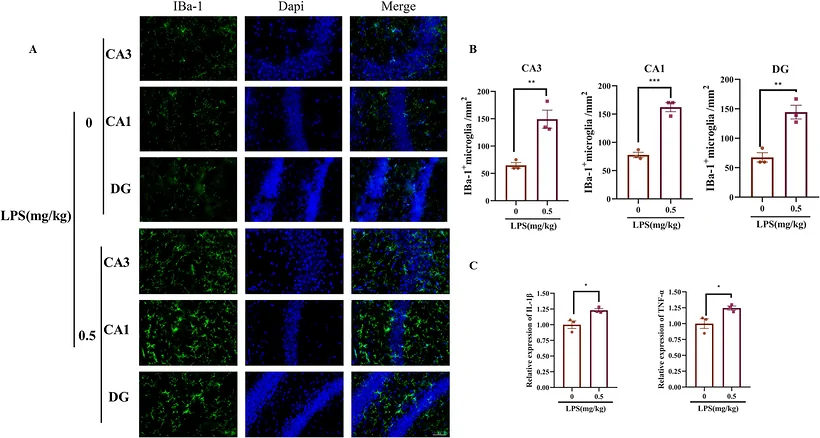
LPS promotes microglial activation and release of pro‐inflammatory factors in the mouse hippocampus. (A) Immunofluorescence results showed the expression of Iba‐1 in hippocampal CA3, CA1, and DG brain regions. Scale bar = 50 µm. (B) The Iba‐1 microglia/mm^2^ values of hippocampal CA3, CA1, and DG brain regions was statistically analyzed. **p < 0.05, **p < 0.01 vs. the Ctrl group. (C) LPS induced a significant increase in the mRNA levels of pro‐inflammatory factors IL‐1β and TNF‐α in the hippocampus (*p < 0.05, **p < 0.01, ***p < 0.001 vs. the Ctrl group, *n* = 3/group).

Microglia play a key role in memory retention and synaptic pruning (Paolicelli et al. 2022). Immunofluorescence staining showed that intraperitoneal injection of LPS increased the number of microglia detected by Iba‐1 staining in different brain regions of the hippocampus (Figure 3A). Quantification results showed LPS‐induced microglial activation in the hippocampus (Figure 3B). The mRNA levels of IL‐1β and TNF‐α were detected. As can be seen in Figure 3C, mice treated with LPS significantly increased the mRNA levels of IL‐1β and TNF‐α (Figure 3C) in the hippocampus.

### 3.4 Minocycline Administration Ameliorates LPS‐Induced Impairment of Scene Fear Memory Consolidation

**FIGURE 4:**
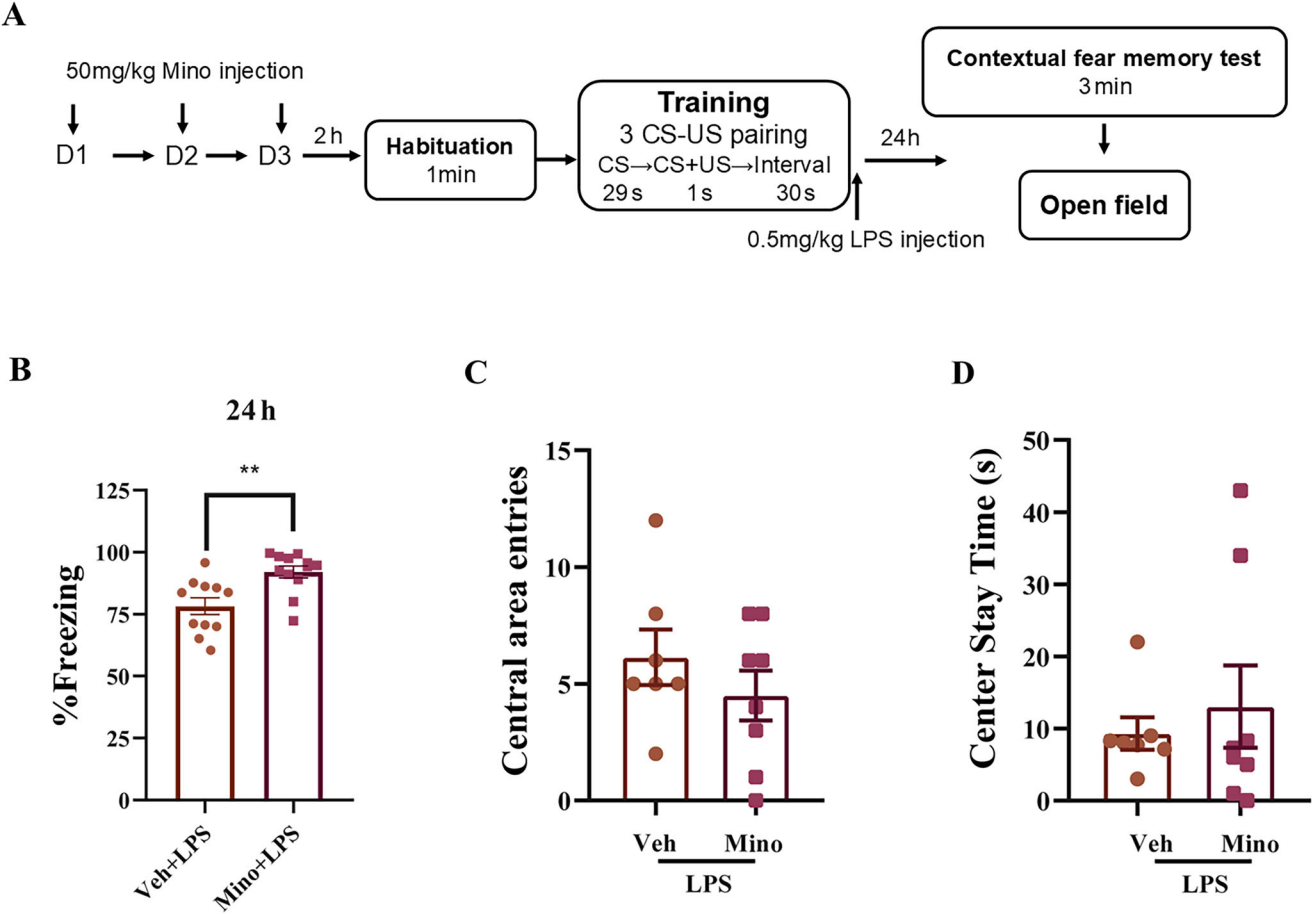
Minocycline treatment ameliorates LPS‐induced impairment of contextual fear memory consolidation in mice. (A) Diagram of the experimental procedure. (B) After pretreatment with minocycline for 3 days, mice received training for fear conditioning. Fear memory was tested 24 h after 0.5 mg/kg LPS treatment. Minocycline treatment significantly alleviated LPS‐induced fear memory impairment. (*n* = 12 for Mino+LPS group and *n* = 11 for the Veh+LPS group) *p < 0.05, **p < 0.01 vs. the Ctrl group. (C, D) Open‐field experiments were performed. The number of central zone entries (C) and the central stay time (D) were measured (*n* = 6/group).

Minocycline has been shown to attenuate LPS‐induced cognitive impairment in mice (Hou et al. 2016). In this study, we examined the effect of treatment with minocycline on LPS‐produced fear memory deficiency. The detailed experimental procedures are shown in Figure 4A, and minocycline treatment significantly improved LPS‐induced impairment of contextual fear memory consolidation (Figure 4B). In addition, the results of the open‐field experiment showed that there was no statistically significant difference in the number of central zone entries and central zone residence time between the two groups (Figure 4C,D), indicating that neither minocycline nor LPS administration had a significant effect on the voluntary activities of mice.

### 3.5 Minocycline Administration Improves LPS‐Induced Elevation of Hippocampal BBB Permeability in Mice

**FIGURE 5:**
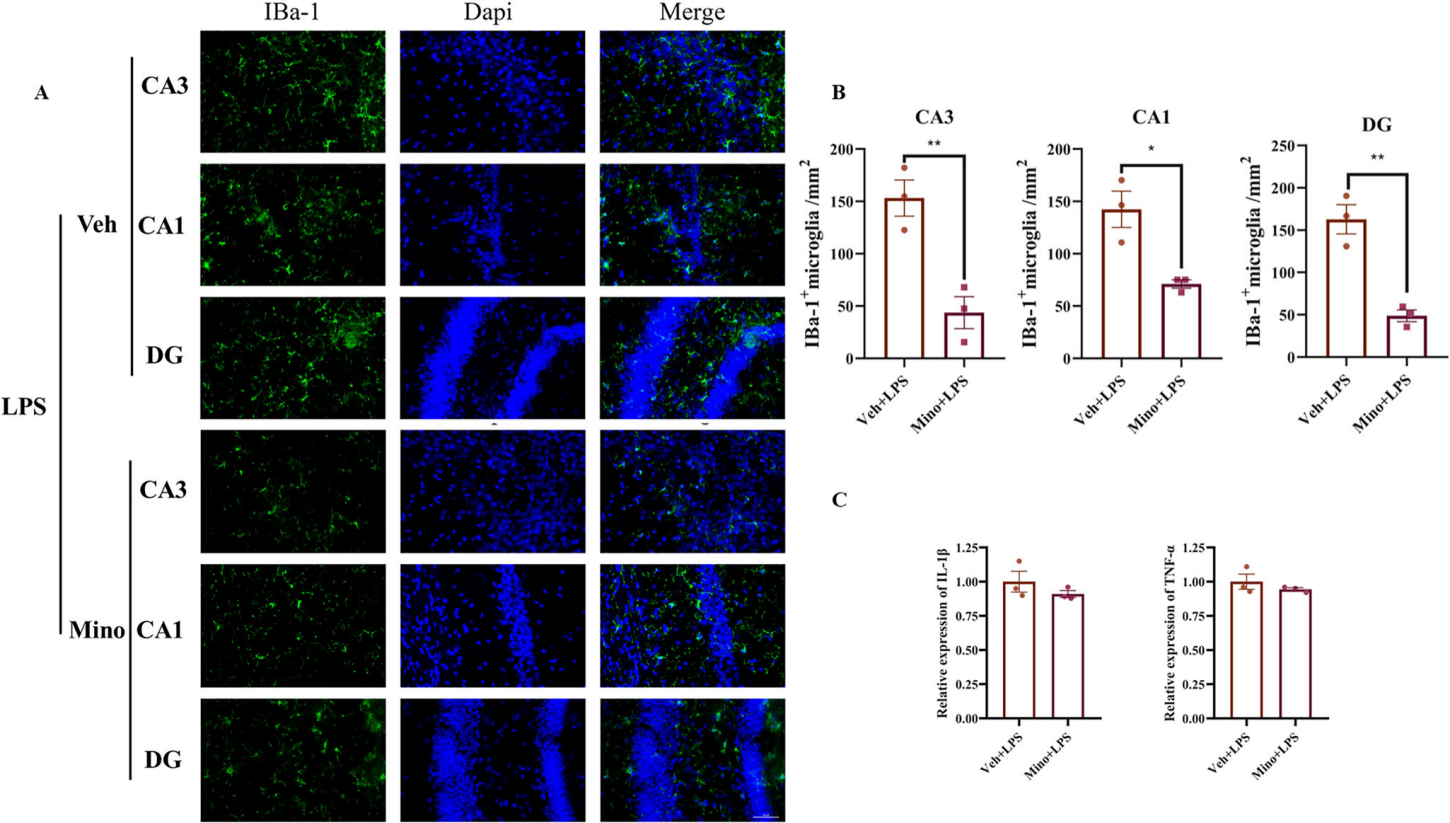
Minocycline administration improves LPS‐induced BBB hyperpermeability in the hippocampus in mice. (A) Immunofluorescence showed the leakage of IgG in the hippocampal CA3, CA1, and DG after minocycline and LPS treatment (*n* = 3/group). Scale bar = 50 µm. (B) Representative Western blot results showed occludin and caveolin‐1 expression after LPS treatment, and minocycline inhibited this effect. (C) The relative band intensity was quantified (*p < 0.05 vs. vehicle, **p < 0.05 vs. the LPS group, *n* = 3/group).

Minocycline is effective in attenuating brain injury caused by transient focal cerebral ischemia due to degradation of the tight junction protein occludin (X. Jin et al. 2013). Immunofluorescence staining was used to explore the effect of minocycline administration on the increased hippocampal BBB permeability induced by LPS. Minocycline significantly decreased LPS‐induced IgG leakage (Figure 5A). Western blot results showed that minocycline significantly decreased LPS‐induced occludin degradation and caveolin‐1 upregulation (Figure 5B,C), suggesting that minocycline administration can improve LPS‐induced BBB hyperpermeability.

### 3.6 Minocycline Administration Inhibits LPS‐Induced Microglial Activation

**FIGURE 6:**
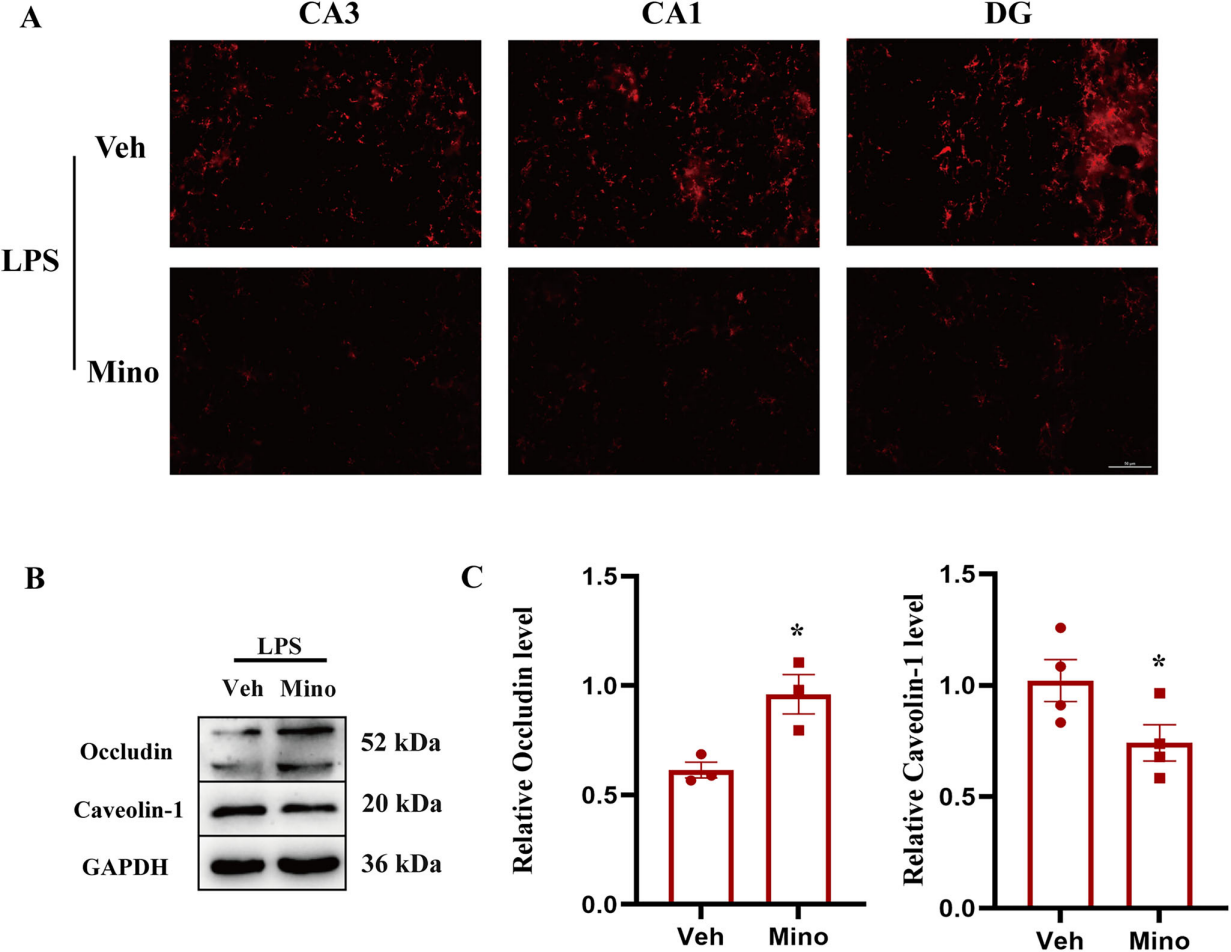
Minocycline treatment inhibits LPS‐induced microglial activation. (A) Immunofluorescence showed the expression of Iba‐1 in hippocampal CA3, CA1, and DG after minocycline and LPS treatment (*n* = 3/group). Scale bar = 50 µm. (B) The Iba‐1 microglia/mm^2^ values was calculated in the CA3, CA1, and DG brain regions. *p < 0.05, **p < 0.01 vs. the Veh LPS group. (C) Although minocycline treatment reduced LPS‐induced increase in mRNA levels of pro‐inflammatory factors IL‐1β and TNF‐α in the hippocampus, there was no significant difference (*p < 0.05 vs the LPS+Veh group; *n* = 3/group).

Minocycline has been shown to cross the BBB and attenuate inflammation associated with microglial activation (G. Wang et al. 2019). To verify whether minocycline alleviates BBB damage by inhibiting microglial activation, immunofluorescence results showed that minocycline treatment significantly decreased LPS‐induced upregulation of the level of Iba‐1 in the hippocampus of mice (Figure 6A, Min+LPS vs. LPS). Quantification results showed that pretreatment with minocycline reduced LPS‐induced microglial activation (Figure 6B). qRT‐PCR results showed that although minocycline decreased LPS‐induced upregulation of pro‐inflammatory factors TNF‐α and IL‐1β, there was no significant difference (Figure 6C).

## 4 Discussion

Appropriate fear responses are adaptive, and disruption of fear memory can lead to an inability to avoid danger (Flores et al. 2018). Fear memory consolidation can be disrupted by various stimuli, including inflammation. Impaired BBB has been shown to play a role in memory deficit; however, it is not clear whether BBB deficit plays a role in LPS‐induced impairment of fear memory consolidation. In the present study, we showed that LPS induced impairment of fear memory consolidation, accompanied by BBB hyperpermeability, microglial activation, and an increase in proinflammatory factors IL‐1β and TNF‐α in the hippocampus. In addition, minocycline treatment can significantly inhibited such effects, suggesting that microglial activation plays a critical role in LPS‐induced contextual fear memory consolidation impairment and BBB disruption.

Systemic inflammation, which releases LPS into circulation (Shukla et al. 2014), can cause emotional and cognitive dysfunction through the release of a substantial quantity of inflammatory factors and the activation of immune cells (Vasconcelos et al. 2014). Our results showed that LPS impaired fear memory consolidation, suggesting a selective disruption of specific hippocampus‐dependent memory function during acute neuroinflammation (Czerniawski et al. 2015). This is consistent with a previous studies showing that LPS administration given after conditioning impaired contextual fear conditioning (Pugh et al. 1998), impaired fear memory consolidation (Kranjac et al. 2012), impaired fear memory reconsolidation in inhibitory avoidance (Shu et al. 2020), impaired spatial memory in the water maze task (Zarifkar et al. 2010), and impaired working memory (Shen et al. 2018; X. Wang et al. 2024).

The hippocampus plays a crucial role in contextual fear memory consolidation. Here, we show that LPS induces BBB damage in the hippocampus. LPS has been shown to impair BBB integrity in depressive‐like behavior (Cao et al. 2025) and in aged mice (X. Wang et al. 2017). Damage to the BBB has been found to lead to cognitive impairment (Montagne et al. 2015), and cognitive function has been improved in a mouse model of vascular cognitive impairment and dementia when PNA5, a novel Mas receptor agonist, was administered, which can reverse BBB permeability damage (Hoyer‐Kimura et al. 2024). Furthermore, aging‐induced episodic‐like memory impairment could be alleviated by melatonin treatment via preserving BBB integrity and upregulating CRTC1 (Y. Wang et al. 2025). Of note, clinical studies have shown that disruption of the hippocampal BBB is an important feature in patients with early cognitive dysfunction (Montagne et al. 2015), and BBB breakdown is critically involved in Alzheimer's disease and vascular disease (Lin et al. 2021).

BBB permeability can be regulated by caveolin‐1 (Andreone et al. 2017; Liu et al. 2012), and caveolin‐1‐mediated BBB disruption via matrix metalloproteases 2/9 contributes to postoperative cognitive dysfunction (Li et al. 2025). In this study, we showed that LPS damages the BBB, accompanied by caveolin‐1 upregulation, and minocycline protects the BBB, accompanied by caveolin‐1 downregulation, suggesting a critical role of caveolin‐1 in LPS‐induced BBB damage and memory impairment.

LPS‐induced inflammation has been demonstrated to cause memory impairment through pro‐inflammatory cytokines (Kranjac et al. 2012; Machado et al. 2015), and the destruction of the BBB is related to inflammation. Microglia have been found to play a key role in memory retention, synaptic pruning, and pathological processes in neurodegenerative diseases (Paolicelli et al. 2022), and microglia can release pro‐inflammatory factors, which, in a pathological state, can impair the integrity of the BBB (Chen et al. 2022; Zhang et al. 2024). Hippocampal‐activated microglia can contribute to BBB impairment and cognitive dysfunction in post‐traumatic stress disorder‐like rats (Ni et al. 2022), while inhibition of microglial activation can maintain the integrity of the BBB (Roseborough et al. 2023). Consistent with a previous study showing that minocycline can protect against LPS‐induced cognitive impairment in mice (Hou et al. 2016), minocycline was found to alleviate LPS‐induced fear memory impairment accompanied by a significant decrease in microglial activation and an improvement in the integrity of the BBB. Although a significant but not large increase in IL‐1β and TNF‐α is still present 24 h after LPS treatment, it is difficult to see a significant decrease in the induction of cytokines at 24 h in the minocycline+LPS group. A very strong LPS‐induced cytokine induction, and perhaps also a strong attenuation by minocycline, could be seen 3–12 h after LPS injection.

### 4.1 Limitation

It should be noted that minocycline is not fully specific for microglia. In addition, when testing the effect of minocycline on LPS‐induced memory impairment, it is important to include a minocycline‐only control group. An experimental design in future studies is warranted to include this drug‐only control group.

## 5 Conclusion

In summary, our results demonstrate that acute exposure to LPS in male mice produced contextual fear memory consolidation impairment, accompanied by BBB hyperpermeability, microglial activation, and the release of the pronflammatory factors IL‐1β and TNF‐α release in the hippocampus. Notably, minocycline treatment significantly inhibited the LPS‐induced detrimental effects.

## Author Contributions

This work was performed and accomplished by all authors. J.Z., L.L., and W.L. contributed to the execution of the entire research project and the statistical analyses. J.Z., W.L., and X.J. wrote the manuscript. All authors have read and approved the final manuscript.

## Funding

This work was supported by the Nature Science Foundation of Shanxi Province (202403021221269).

## Ethics Statement

The study was approved by the Animal Care Committee of Capital Medical University (AEEI‐2023‐014) and was carried out following the NIH Guide for the Care and Use of Laboratory Animals.

## Conflicts of Interest

The authors declare no conflicts of interest.

## Data Availability

The data that support the findings of this study are available from the corresponding author upon reasonable request.

## References

[brb371747-bib-0001] Andreone, B. J. , B. W. Chow , A. Tata , et al. 2017. “Blood‐Brain Barrier Permeability Is Regulated by Lipid Transport‐Dependent Suppression of Caveolae‐Mediated Transcytosis.” Neuron 94, no. 3: 581–594.e5. 10.1016/j.neuron.2017.03.043. 28416077 PMC5474951

[brb371747-bib-0002] Cao, M. M. , Z. Guo , J. Wang , et al. 2025. “Astragalin Alleviates Lipopolysaccharide‐Induced Depressive‐Like Behavior in Mice by Preserving Blood‐Brain Barrier Integrity and Suppressing Neuroinflammation.” Free Radical Biology and Medicine 232: 340–352. 10.1016/j.freeradbiomed.2025.03.014. 40089077

[brb371747-bib-0003] Chen, S. , Y. Sun , F. Li , et al. 2022. “Modulation of α7nAchR by Melatonin Alleviates Ischemia and Reperfusion‐Compromised Integrity of Blood–Brain Barrier Through Inhibiting HMGB1‐Mediated Microglia Activation and CRTC1‐Mediated Neuronal Loss.” Cellular and Molecular Neurobiology 42, no. 7: 2407–2422. 10.1007/s10571-021-01122-2. 34196879 PMC11421614

[brb371747-bib-0004] Czerniawski, J. , T. Miyashita , G. Lewandowski , and J. F. Guzowski . 2015. “Systemic Lipopolysaccharide Administration Impairs Retrieval of Context–Object Discrimination, but Not Spatial, Memory: Evidence for Selective Disruption of Specific Hippocampus‐Dependent Memory Functions During Acute Neuroinflammation.” Brain, Behavior, and Immunity 44: 159–166. 10.1016/j.bbi.2014.09.014. 25451612 PMC4358899

[brb371747-bib-0005] Davis, A. C. , E. J. Goodman , L. M. Wangler , et al. 2026. “Interleukin‐1 Receptor‐1 Signaling Mediates Neuroinflammation, Neuronal Injury, and Cognitive Decline After Diffuse Traumatic Brain Injury.” Brain, Behavior, and Immunity 135: 106524. 10.1016/j.bbi.2026.106524. 41780806 PMC13555749

[brb371747-bib-0006] Eyvari‐Brooshghalan, S. , R. Haddadi , S. Shahidi , et al. 2025. “Acute Treatment With Fucoidan Ameliorates Traumatic Brain Injury‐Induced Neurological Damages and Memory Deficits in Rats: Role of BBB Integrity, Microglial Activity, Neuroinflammation, and Oxidative Stress.” Molecular Neurobiology 62, no. 5: 5990–6013. 10.1007/s12035-024-04668-6. 39692820

[brb371747-bib-0007] Flores, A. , M. A. Fullana , C. Soriano‐Mas , and R. Andero . 2018. “Lost in Translation: How to Upgrade Fear Memory Research.” Molecular Psychiatry 23: 2122–2132. 10.1038/s41380-017-0006-0. 29298989

[brb371747-bib-0008] Haskel, M. V. L. , L. S. da Silva Vequi , M. W. P. Flauzino , et al. 2026. “Taurine Reverses Long‐Term Memory Deficits Caused by LPS Induced Neuroinflammation.” BMC Neuroscience 27: 30. 10.1186/s12868-026-01017-2. 42277642 PMC13483688

[brb371747-bib-0009] Haubrich, J. , M. Bernabo , A. G. Baker , and K. Nader . 2020. “Impairments to Consolidation, Reconsolidation, and Long‐Term Memory Maintenance Lead to Memory Erasure.” Annual Review of Neuroscience 43: 297–314. 10.1146/annurev-neuro-091319-024636. 32097575

[brb371747-bib-0010] Hou, Y. , G. Xie , X. Liu , et al. 2016. “Minocycline Protects Against Lipopolysaccharide‐Induced Cognitive Impairment in Mice.” Psychopharmacology 233, no. 5: 905–916. 10.1007/s00213-015-4169-6. 26645224

[brb371747-bib-0011] Hoyer‐Kimura, C. , M. Hay , J. P. Konhilas , et al. 2024. “PNA5, A Novel Mas Receptor Agonist, Improves Neurovascular and Blood‐Brain‐Barrier Function in a Mouse Model of Vascular Cognitive Impairment and Dementia.” Aging and Disease 15, no. 4: 1927–1951. 37815905 10.14336/AD.2023.0928 PMC11272189

[brb371747-bib-0012] Hu, X. , J. Dong , P. Geng , et al. 2024. “Nicotine Treatment Ameliorates Blood‐Brain Barrier Damage After Acute Ischemic Stroke by Regulating Endothelial Scaffolding Protein Pdlim5.” Translational Stroke Research 15, no. 3: 672–687. 10.1007/s12975-023-01158-0. 37233908

[brb371747-bib-0013] Jin, X. , J. Liu , K. J. Liu , G. A. Rosenberg , Y. Yang , and W. Liu . 2013. “Normobaric Hyperoxia Combined With Minocycline Provides Greater Neuroprotection Than Either Alone in Transient Focal Cerebral Ischemia.” Experimental Neurology 240: 9–16. 10.1016/j.expneurol.2012.11.018. 23195595 PMC3563388

[brb371747-bib-0014] Jin, X. C. , Y. F. Lu , X. F. Yang , L. Ma , and B. M. Li . 2007. “Glucocorticoid Receptors in the Basolateral Nucleus of Amygdala Are Required for Postreactivation Reconsolidation of Auditory Fear Memory.” European Journal of Neuroscience 25, no. 12: 3702–3712. 10.1111/j.1460-9568.2007.05621.x. 17610589

[brb371747-bib-0015] Kranjac, D. , K. A. McLinden , L. E. Deodati , M. R. Papini , M. J. Chumley , and G. W. Boehm . 2012. “Peripheral Bacterial Endotoxin Administration Triggers Both Memory Consolidation and Reconsolidation Deficits in Mice.” Brain, Behavior, and Immunity 26, no. 1: 109–121. 10.1016/j.bbi.2011.08.005. 21889586

[brb371747-bib-0016] Li, X. , W. Ding , X. Zhang , et al. 2025. “Caveolin‐1–Mediated Blood‐Brain Barrier Disruption via MMP2/9 Contributes to Postoperative Cognitive Dysfunction.” Neurochemical Research 50, no. 4: 210. 10.1007/s11064-025-04458-z. 40571858 PMC12202573

[brb371747-bib-0017] Lin, Z. , S. Sur , P. Liu , et al. 2021. “Blood–Brain Barrier Breakdown in Relationship to Alzheimer and Vascular Disease.” Annals of Neurology 90, no. 2: 227–238. 10.1002/ana.26134. 34041783 PMC8805295

[brb371747-bib-0018] Liu, J. , X. Jin , K. J. Liu , and W. Liu . 2012. “Matrix Metalloproteinase‐2‐Mediated Occludin Degradation and Caveolin‐1‐Mediated Claudin‐5 Redistribution Contribute to Blood–Brain Barrier Damage in Early Ischemic Stroke Stage.” Journal of Neuroscience 32, no. 9: 3044–3057. 10.1523/JNEUROSCI.6409-11.2012. 22378877 PMC3339570

[brb371747-bib-0019] Machado, I. , P. V. Gonzalez , A. Vilcaes , et al. 2015. “Interleukin‐1β‐Induced Memory Reconsolidation Impairment Is Mediated by a Reduction in Glutamate Release and zif268 Expression and α‐Melanocyte‐Stimulating Hormone Prevented These Effects.” Brain, Behavior, and Immunity 46: 137–146. 10.1016/j.bbi.2015.01.012. 25637483

[brb371747-bib-0020] Mao, M. , Z. Zhou , M. Sun , C. Wang , and J. Sun . 2021. “The Dysfunction of Parvalbumin Interneurons Mediated by Microglia Contributes to Cognitive Impairment Induced by Lipopolysaccharide Challenge.” Neuroscience Letters 762: 136133. 10.1016/j.neulet.2021.136133. 34311051

[brb371747-bib-0021] Montagne, A. , S. R. Barnes , M. D. Sweeney , et al. 2015. “Blood‐Brain Barrier Breakdown in the Aging Human Hippocampus.” Neuron 85, no. 2: 296–302. 10.1016/j.neuron.2014.12.032. 25611508 PMC4350773

[brb371747-bib-0022] Ni, K. , J. Zhu , X. Xu , et al. 2022. “Hippocampal Activated Microglia May Contribute to Blood–Brain Barrier Impairment and Cognitive Dysfunction in Post‐Traumatic Stress Disorder‐Like Rats.” Journal of Molecular Neuroscience 72, no. 5: 975–982. 10.1007/s12031-022-01981-4. 35167061 PMC8852956

[brb371747-bib-0023] Paolicelli, R. C. , A. Sierra , B. Stevens , et al. 2022. “Microglia States and Nomenclature: A Field at Its Crossroads.” Neuron 110, no. 21: 3458–3483. 10.1016/j.neuron.2022.10.020. 36327895 PMC9999291

[brb371747-bib-0024] Pugh, C. R. , K. Kumagawa , M. Fleshner , L. R. Watkins , S. F. Maier , and J. W. Rudy . 1998. “Selective Effects of Peripheral Lipopolysaccharide Administration on Contextual and Auditory‐Cue Fear Conditioning.” Brain, Behavior, and Immunity 12, no. 3: 212–229. 10.1006/brbi.1998.0524. 9769157

[brb371747-bib-0025] Roseborough, A. D. , Y. Zhu , L. Zhao , S. R. Laviolette , S. H. Pasternak , and S. N. Whitehead . 2023. “Fibrinogen Primes the Microglial NLRP3 Inflammasome and Propagates Pro‐Inflammatory Signaling via Extracellular Vesicles: Implications for Blood‐Brain Barrier Dysfunction.” Neurobiology of Disease 177: 106001. 10.1016/j.nbd.2023.106001. 36646389

[brb371747-bib-0026] Shen, X. , Y. Sun , M. Wang , et al. 2018. “Chronic N‐Acetylcysteine Treatment Alleviates Acute Lipopolysaccharide‐Induced Working Memory Deficit Through Upregulating Caveolin‐1 and Synaptophysin in Mice.” Psychopharmacology 235, no. 1: 179–191. 10.1007/s00213-017-4762-y. 29058042

[brb371747-bib-0027] Shu, H. , M. Wang , M. Song , et al. 2020. “Acute Nicotine Treatment Alleviates LPS‐Induced Impairment of Fear Memory Reconsolidation Through AMPK Activation and CRTC1 Upregulation in Hippocampus.” International Journal of Neuropsychopharmacology 23, no. 10: 687–699. 10.1093/ijnp/pyaa043. 32516360 PMC7727489

[brb371747-bib-0028] Shukla, P. , G. M. Rao , G. Pandey , et al. 2014. “Therapeutic Interventions in Sepsis: Current and Anticipated Pharmacological Agents.” British Journal of Pharmacology 171, no. 22: 5011–5031. 10.1111/bph.12829. 24977655 PMC4253453

[brb371747-bib-0029] Skelly, D. T. , É. W. Griffin , C. L. Murray , et al. 2018. “Acute Transient Cognitive Dysfunction and Acute Brain Injury Induced by Systemic Inflammation Occur by Dissociable IL‐1‐Dependent Mechanisms.” Molecular Psychiatry 24: 1533–1548. 10.1038/s41380-018-0075-8. 29875474 PMC6510649

[brb371747-bib-0030] Vasconcelos, A. R. , L. M. Yshii , T. A. Viel , et al. 2014. “Intermittent Fasting Attenuates Lipopolysaccharide‐Induced Neuroinflammation and Memory Impairment.” Journal of Neuroinflammation 11: 85. 10.1186/1742-2094-11-85. 24886300 PMC4041059

[brb371747-bib-0031] Vasudevan, K. , J. E. Hassell Jr. , and S. Maren . 2024. “Hippocampal Engrams and Contextual Memory.” Advances in Neurobiology 38: 45–66. 10.1007/978-3-031-62983-910.1007/978-3-031-62983-9_4. 39008010 PMC12006847

[brb371747-bib-0032] Wang, G. , Z. Li , S. Li , et al. 2019. “Minocycline Preserves the Integrity and Permeability of BBB by Altering the Activity of DKK1–Wnt Signaling in ICH Model.” Neuroscience 415: 135–146. 10.1016/j.neuroscience.2019.06.038. 31344398

[brb371747-bib-0033] Wang, Q. , M. W. Wang , Y. Y. Sun , et al. 2023. “Nicotine Pretreatment Alleviates MK‐801‐Induced Behavioral and Cognitive Deficits in Mice by Regulating Pdlim5/CRTC1 in the PFC.” Acta Pharmacologica Sinica 44, no. 4: 780–790. 10.1038/s41401-022-00974-8. 36038765 PMC10042998

[brb371747-bib-0034] Wang, X. , Y. Liu , Y. Sun , W. Liu , and X. Jin . 2016. “Blood Brain Barrier Breakdown Was Found in Non‐Infarcted Area After 2‐h MCAO.” Journal of the Neurological Sciences 363: 63–68. 10.1016/j.jns.2016.02.035. 27000223

[brb371747-bib-0035] Wang, X. , Q. Wang , M. Song , et al. 2024. “Chronic but Not Acute Nicotine Treatment Ameliorates Acute Inflammation‐Induced Working Memory Impairment by Increasing CRTC1 and HCN2 in Adult Male Mice.” CNS Neuroscience & Therapeutics 30, no. 2: e14627. 10.1111/cns.14627. 38353058 PMC10865150

[brb371747-bib-0036] Wang, X. , G. X. Xue , W. C. Liu , et al. 2017. “Melatonin Alleviates Lipopolysaccharide‐Compromised Integrity of Blood‐Brain Barrier Through Activating AMP‐Activated Protein Kinase in Old Mice.” Aging Cell 16, no. 2: 414–421. 10.1111/acel.12572. 28156052 PMC5334533

[brb371747-bib-0037] Wang, Y. , W. Du , Y. Sun , J. Zhang , C. Ma , and X. Jin . 2023. “CRTC1 is a Potential Target to Delay Aging‐Induced Cognitive Deficit by Protecting the Integrity of the Blood‐Brain Barrier via Inhibiting Inflammation.” Journal of Cerebral Blood Flow & Metabolism 43, no. 7: 1042–1059. 10.1177/0271678X231169133. 37086081 PMC10291461

[brb371747-bib-0038] Wang, Y. , X. Zhang , H. Guo , et al. 2025. “Aging‐Induced Episodic‐Like Memory Impairment Could be Alleviated by Melatonin Treatment via Preserving Blood–Brain Barrier Integrity and Upregulating CRTC1.” CNS Neuroscience & Therapeutics 31, no. 4: e70412. 10.1111/cns.70412. 40285336 PMC12031892

[brb371747-bib-0039] Wang, Y. P. , W. H. Du , X. Y. Hu , et al. 2023. “Targeting the Blood–Brain Barrier to Delay Aging‐Accompanied Neurological Diseases by Modulating Gut Microbiota, Circadian Rhythms, and Their Interplays.” Acta Pharmaceutica Sinica B 13, no. 12: 4667–4687. 10.1016/j.apsb.2023.08.009. 38045038 PMC10692395

[brb371747-bib-0040] Wei, P. , Q. Liu , D. Li , Q. Zheng , J. Zhou , and J. Li . 2015. “Acute Nicotine Treatment Attenuates Lipopolysaccharide‐Induced Cognitive Dysfunction by Increasing BDNF Expression and Inhibiting Neuroinflammation in the Rat Hippocampus.” Neuroscience Letters 604: 161–166. 10.1016/j.neulet.2015.08.008. 26259694

[brb371747-bib-0041] Zarifkar, A. , S. Choopani , R. Ghasemi , et al. 2010. “Agmatine Prevents LPS‐Induced Spatial Memory Impairment and Hippocampal Apoptosis.” European Journal of Pharmacology 634, no. 1–3: 84–88. 10.1016/j.ejphar.2010.02.029. 20184876

[brb371747-bib-0042] Zhang, X. L. , W. H. Du , S. X. Qian , et al. 2024. “Glial Growth Factor 2 Treatment Alleviates Ischemia and Reperfusion‐Damaged Integrity of the Blood‐Brain Barrier Through Decreasing Mfsd2a/Caveolin‐1‐Mediated Transcellular and Pdlim5/YAP/TAZ‐Mediated Paracellular Permeability.” Acta Pharmacologica Sinica 45, no. 11: 2241–2252. 10.1038/s41401-024-01323-7. 38902501 PMC11489722

